# A Novel Genetic Group of Bovine* Hepacivirus* in Archival Serum Samples from Brazilian Cattle

**DOI:** 10.1155/2017/4732520

**Published:** 2017-08-20

**Authors:** Cláudio W. Canal, Matheus N. Weber, Samuel P. Cibulski, Mariana S. Silva, Daniela E. Puhl, Hanspeter Stalder, Ernst Peterhans

**Affiliations:** ^1^Laboratório de Virologia, Faculdade de Veterinária, Universidade Federal do Rio Grande do Sul (UFRGS), Porto Alegre, RS, Brazil; ^2^Institute of Veterinary Virology, University of Bern, Bern, Switzerland

## Abstract

Hepatitis C virus (HCV) (genus* Hepacivirus*; family Flaviviridae) is a major human pathogen causing persistent infection and hepatic injury. Recently, emerging HCV-like viruses were described infecting wild animals, such as bats and rodents, and domestic animals, including dogs, horses, and cattle. Using degenerate primers for detecting bovine pestiviruses in a 1996 survey three bovine serum samples showed a low identity with the genus* Pestivirus* of the Flaviviridae family. A virus could not be isolated in cell culture. The description of bovine hepaciviruses (BovHepV) in 2015 allowed us to retrospectively identify the sequences as BovHepV, with a 88.9% nucleotide identity. In a reconstructed phylogenetic tree, the Brazilian BovHepV samples grouped within the bovine HCV-like cluster in a separated terminal node that was more closely related to the putative bovine* Hepacivirus* common ancestor than to bovine hepaciviruses detected in Europe and Africa.

## 1. Introduction

Hepatitis C virus (HCV) is an enveloped single stranded RNA virus that represents the type species of the* Hepacivirus* genus within the Flaviviridae family. Its genome with a length of about 9.6 kb contains two untranslated regions (UTR) at the 5′ and 3′ ends. Hepaciviruses differ from the other Flaviviridae genera,* Flavivirus* and* Pestivirus,* by their limited multiplication in cultured cells [1].

HCV represent one of the most significant threats to human health leading to hepatitis, liver cirrhosis, and hepatocellular carcinoma [2, 3]. Currently, about 160 million individuals are persistently infected with HCV [4]. Acute HCV infection is asymptomatic in many cases, but 50–80% of infected individuals are unable to clear the virus leading to a state of persistent viral replication and hepatic inflammation takes place [2, 3].

Recently, novel HCV-related hepaciviruses were detected in nonprimate hosts. In 2011, HCV-like viruses were reported in dogs displaying signs of hepatic injury [5] and respiratory illness [6]. Further investigations revealed that the natural reservoirs of this HCV-like virus are not dogs but horses [5, 7] and that it was not associated with liver disease in dogs [8]. These strains were designated nonprimate hepaciviruses (NPHV) [7, 9]. Moreover, a great diversity of* Hepacivirus* sequences was detected in rodents and bats worldwide [10–12].

These previous reports highlight the importance of the search for possible new* Hepacivirus* reservoirs and of investigating the risks arising for public health. In 2015, a study performed in Germany reported HCV-like viruses that showed liver tropism and chronic infection in domestic cattle without any signs of clinical disease [13]. Another study performed in Africa also found closely related viruses [14]. In the present study we describe and characterize a bovine* Hepacivirus* (BovHepV) detected in cattle from Southern Brazil during a BVDV survey that emphasizes a possible worldwide spread of this emerging group of viruses.

## 2. Methods

Six bovine serum samples from a farm located in Carazinho, Rio Grande do Sul State, Southern Brazil, were collected in 1996 when screening for bovine viral diarrhea virus (BVDV). The RNA was isolated using TRIzol® LS Reagent (Invitrogen, Carlsbad, CA, USA) in a total volume of 250 *μ*L, according to the manufacturer's instructions. The cDNA was synthetized using SuperScript® II (Invitrogen) according to the manufacturer's recommendation.

For PCR, we used the reverse primer 326 previously described by Vilček et al. with a final concentration of 0.6 *μ*M [15]. As forward primers, we used a mixture of the eight different primers (Table 1) which were mixed at equal amounts to give a final concentration of 0.6 *μ*M [16]. The forward primers bind approximately at position 52–76 and the reverse primer at position 284–304 in the BovHepV-B1/Ger/213 strain (GenBank accession number KP641123) (Table 1).

PCR amplification products were purified using Illustra GFX PCR DNA and Gel Band Purification Kit (GE Healthcare Life Sciences, Uppsala, Sweden), and both strands were sequenced three times with an ABI PRISM 3100 Genetic Analyzer (Applied Biosystems) using a BigDye Terminator v.3.1 cycle sequencing kit (Applied Biosystems). The sequences were assembled using Geneious software version 8.1.4 (Biomatters, Auckland, New Zealand). The sequences detected in the present study were deposited in GenBank under accession numbers KY439906–KY439908.

Sequences of 24 hepaciviruses, including reference and representative strains, were retrieved from GenBank (https://www.ncbi.nlm.nih.gov/genbank/) and aligned using MUSCLE software [17]. Phylogenetic trees were reconstructed with MrBayes v3.2.1 [18] using Bayesian analysis coupled with Markov Chain Monte Carlo methods of phylogenetic inference. For Bayesian analysis, the Jukes-Cantor model was chosen using jModeltest and used as the substitution model (rates variation across sites invariable + gamma). Markov Chain Monte Carlo chains were run for 1,100,000 generations, sampling every 100 generations, and the first 100,000 sampled trees were discarded as burn-in. Trees obtained before convergent and stable likelihood values were discarded (i.e., the 100,000 first generations were burn-in).

RT-PCR-positive serum samples were submitted to virus isolation in cell culture using the cell lines Madin Darby bovine kidney (MDBK) (ATCC® CCL-22™), baby hamster kidney 21 (BHK-21) (ATCC CCL-10™), rabbit kidney 13 (RK-13) (ATCC CCL-37™), mouse fibroblast NCTC clone 929 (L929) (ATCC CCL-1™), and bovine testicle and bovine turbinate primary cell cultures. The cells were grown in minimal essential medium (MEM), supplemented with L-glutamine (1.4 mM), gentamicin (50 mg/liter), and 10% fetal bovine serum (FBS). For virus isolation, 25 cm^2^ flasks containing 70% confluent cell monolayers were inoculated with serum samples and incubated at 37°C for 72 to 96 h. Following one freeze-thaw cycle, the suspension was centrifuged for 10 min at 1,000 ×g. Supernatants were collected, aliquoted, and submitted to two more passages in the cell lines followed by RT-PCR as above described to verify the virus presence.

## 3. Results and Discussion

During a 1996 survey of bovine pestiviruses, using degenerate primers aiming to amplify bovine pestiviruses, three bovine serum samples were positive by RT-PCR. The sequenced amplification product showed a low identity with the known Flaviviridae members. Importantly, the sequences were unrelated to known pestiviruses. However, a retrospective comparison revealed that the three 217 bp amplicons were 88.9% identical with the recently discovered bovine hepaciviruses (BovHepV) [13, 14].

Remarkably, the RT-PCR protocol developed for detecting bovine pestiviruses in our study successfully amplified 5′UTR of BovHepV. It is important to point out that the primers used by us were modified [16] from those commonly used to amplify 5′UTR of pestiviruses [15]. The 5′UTR of the Flaviviridae members contains an internal ribosome entry site (IRES) and is conserved [1], providing an opportunity for adaptation of protocols to detect novel viruses.

**Figure 1:**
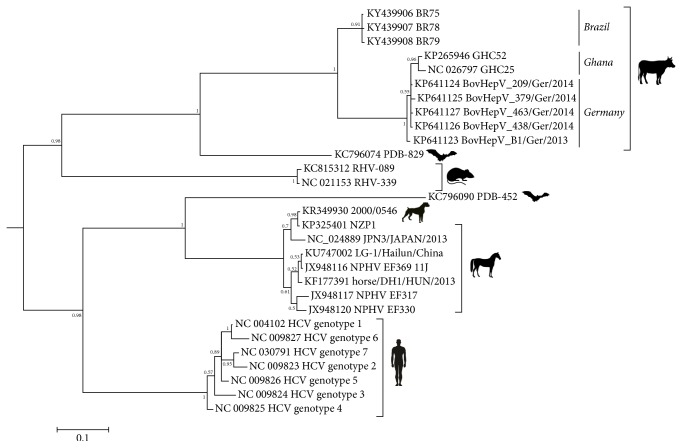
*Phylogenetic tree of 5*′*UTR of hepaciviruses*. Sequences from Brazil (BR75, BR78, and BR79) and representative hepaciviruses strains were analyzed by Bayesian analysis coupled with Markov Chain Monte Carlo methods of phylogenetic inference. The length of each pair of branches represents the distance between sequence pairs in the rectangular tree. The scale bar represents the percentage of nucleotide differences in the rectangular tree.

The Bayesian phylogenetic tree (Figure 1), reconstructed with the Brazilian BovHepV and representative strains within the genus, presented two well separated branches supported by posterior probability values of 1, where bovine and rodent hepaciviruses grouped in the same branch, and HCV, bat, canine, and equine hepaciviruses in the other. BR75, BR78, and BR79 grouped in the bovine BovHepV cluster supported by a posterior probability value of 1 but were located in a separated terminal node that was more closely related to a putative BovHepV common ancestor than to the viruses detected in Europe and Africa [13, 14]. The data presented here showed that Brazilian BovHepV diverge from European and African strains and suggest that variants of BovHepV may circulate in cattle worldwide. None of the cell cultures used in our study for the isolation of BR75, BR78, and BR79 supported the multiplication of BovHepV, which supports earlier observations of a limited capacity of hepaciviruses to grow in cultured cells [1, 19, 20]. Only one single HCV strain, JFH1, has been found to efficiently infect cultured cells, and this was the case only in a human hepatoma cell line (Huh7) [19]. Although the nucleotide sequences reported here do not comprise full genomes, the data suggest that BovHepV may have circulated in Brazilian cattle at least in 1996, some 20 years before those described in Germany and Africa [13, 14]. The sequences of the Brazilian BovHepV differ significantly from those of the previously described viruses, which suggests that hepaciviruses of cattle may be more diverse than originally assumed. It is important to highlight that it is the first report of BovHepV in Brazilian cattle.

Finally, it is important to note that the Brazilian BovHepV described in this work were detected when screening cattle sera for BVDV using primers designed to detect a broader spectrum of bovine pestiviruses [16]. This highlights the genetic relationship between the Flaviviridae members in 5′UTR. FBS is used in media for growing viruses in cultured cells and is known to be a risk factor for spreading BVDV, especially in live vaccines [21–23]. Keeping in mind the diverse mechanisms of high genetic plasticity of RNA viruses, including recombination, it may be of interest to extend the screening protocols for adventitious agents in FBS to BovHepV.

## Figures and Tables

**Table 1:** Nucleotide sequence of the primers used for RT-PCR.

Primer	Sequence (5′ to 3′)
230a	TAG CCA TGC CCT TAG TAG GAC TAG C
230b	TAG CCA TGC CCT TAG TAG GAC AAG C
230c	TAG CCA TGC CCT TAG TAG GAG TAG C
230d	TAA CCA TAC CCT TAG TAG GAC TAG C
230e	TAG CCA TAC CCG TAG TAG GAC TAG C
230f	TAG CCA TAC ACG TAG TAG GAC TAG C
230g	TAG CCA TGC CCA TAG TAG GAC TAG C
230h	TAG CCA TGC CCA CAG TAG GAC TAG C
